# Ex vivo blockade of PI3K gamma or delta signaling enhances the antitumor potency of adoptively transferred CD8^+^ T cells

**DOI:** 10.1002/eji.201948455

**Published:** 2020-05-28

**Authors:** Connor J. Dwyer, Dimitrios C. Arhontoulis, Guillermo O Rangel Rivera, Hannah M. Knochelmann, Aubrey S. Smith, Megan M. Wyatt, Mark P. Rubinstein, Carl Atkinson, Jessica E. Thaxton, David M. Neskey, Chrystal M. Paulos

**Affiliations:** ^1^ Department of Microbiology and Immunology Medical University of South Carolina Charleston SC USA; ^2^ Department of Dermatology and Dermatologic Surgery Medical University of South Carolina Charleston SC USA; ^3^ Department of Surgery Medical University of South Carolina Charleston SC USA; ^4^ Department of Surgery Transplant Immunobiology Laboratory Medical University of South Carolina Charleston SC USA; ^5^ Department of Orthopedics Medical University of South Carolina Charleston SC USA; ^6^ Department of Otolaryngology Head and Neck Surgery Medical University of South Carolina Charleston SC USA; ^7^ Department of Cell and Molecular Pharmacology and Developmental Therapeutics Medical University of South Carolina Charleston SC USA

**Keywords:** ACT, Cancer, CAR, Phosphoinositide 3‐kinase, T cells

## Abstract

Adoptive T cell transfer therapy induces objective responses in patients with advanced malignancies. Despite these results, some individuals do not respond due to the generation of terminally differentiated T cells during the expansion protocol. As the gamma and delta catalytic subunits in the PI3K pathway are abundant in leukocytes and involved in cell activation, we posited that blocking both subunits ex vivo with the inhibitor IPI‐145 would prevent their differentiation, thereby increasing antitumor activity in vivo. However, IPI‐145 treatment generated a product with reduced antitumor activity. Instead, T cells inhibited of PI3Kγ (IPI‐549) or PI3Kδ (CAL‐101 or TGR‐1202) alone were more potent in vivo. While T cells coinhibited of PI3Kγ and PI3Kδ were less differentiated, they were functionally impaired, indicated by reduced production of effector cytokines after antigenic re‐encounter and decreased persistence in vivo. Human CAR T cells expanded with either a PI3Kγ or PI3Kδ inhibitor possessed a central memory phenotype compared to vehicle cohorts. We also found that PI3Kδ‐inhibited CARs lysed human tumors in vitro more effectively than PI3Kγ‐expanded or traditionally expanded CAR T cells. Our data imply that sole blockade of PI3Kγ or PI3Kδ generates T cells with remarkable antitumor properties, a discovery that has substantial clinical implications.

## Graphical abstract

Current expansion protocols generate T cells yields with reduced antitumor immunity. We demonstrate that incorporation of PI3K or PI3K inhibitors during expansion generates more therapeutic T cell products against solid tumors. Yet, co‐blockade of both PI3K and PI3K ex vivo generated T cells that were less effective in vivo.

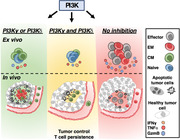

## Introduction

CD19‐specific chimeric antigen receptor (CAR) T cell therapy mediates potent responses in patients with certain hematological malignancies [1, 2, 3]. Yet, CAR T cell therapy has been less successful in patients with solid malignancies [4, 5]. Complications with this form of therapy, known as adoptive T cell transfer (ACT) therapy, arise from the current manufacturing protocols used to generate antitumor T cells, sometimes relying on multiple rounds of antigen‐specific stimulation and IL‐2, which drive T cells to a terminally differentiated state [6, 7]. While these FDA‐approved protocols generate ample cells, they do not persist [8, 9, 10]. Emerging preclinical work shows that less differentiated stem or central memory T cells persist and can effectively clear tumors [11, 12, 13]. Moreover, stem memory T cells have been reported as the lymphocyte population receptive in patients to PD‐1 blockade therapy [14].

To uncouple T cell differentiation from expansion, drugs that inhibit downstream mediators of the TCR signaling pathway have been incorporated into ex vivo expansion protocols [15, 16, 17]. One such target is the phosphoinositide 3‐kinase (PI3K) family which comprises of multisubunit kinases that mediate essential roles in immune cell proliferation, differentiation, and metabolism [18, 19, 20, 21, 22]. Class I PI3Ks convert PI(4,5)P2 to PI(3,4,5)P3, which acts as a secondary signal to activate AKT signaling [23]. PI3Kα and PI3Kβ are expressed ubiquitously while PI3Kγ and PI3Kδ are primarily associated with immune cells [24, 25].

Hyperactive PI3K activity is a hallmark of hematological malignancies, warranting the use of small molecule inhibitors to kill cancer cells [26]. Idelalisib (CAL‐101), a PI3Kδ inhibitor, is the only FDA approved PI3K inhibitor to treat hematological malignancies. In some instances, oral administration of CAL‐101 to patients was discontinued due to severe gastrointestinal toxicities and infection [27, 28]. Current efforts have focused on synthesizing new inhibitors that are effective but less toxic. TGR‐1202 is a next generation PI3Kδ inhibitor (that also blocks casein kinase 1ε) showing promise in patients but with reduced adverse events compared to those administered CAL‐101 [29, 30]. IPI‐145, a drug that blocks both PI3Kγ and PI3Kδ, has also shown promise, with nominal toxicity in a small cohort of patients with advanced hematological malignancies [31, 32]. As Tregs and B cells are dependent on PI3Kδ, this catalytic subunit is commonly targeted [18, 22]. However, PI3Kγ inhibition has been used to remove suppressive myeloid immune cells in the tumor [33, 34]. IPI‐549, a PI3Kγ inhibitor, in early‐stage clinical trials is used to treat patients with solid tumors.

In order to avoid in vivo toxicity, our group reported that CAL‐101 can be used ex vivo to generate powerful antitumor T cells [35, 36]. T cells treated with CAL‐101 were less differentiated ex vivo and could persist with improved immunity in vivo. While PI3Kδ blockade augments cell products, it is unknown whether PI3Kγ inhibition can produce similarly therapeutic T cell products [19, 37]. As PI3Kγ and PI3Kδ are uniquely expressed in immune cells, we sought to test the impact of drugs that inhibit one or both subunits in T cells. Data presented here reveal that ex vivo inhibition of either PI3Kγ or PI3Kδ, but not simultaneous inhibition of both subunits, generates T cells with enhanced therapeutic capabilities in vivo. Additional investigation revealed that PI3Kδ blockade was more effective than PI3Kγ inhibition for the generation of human CAR T cells with enhanced lytic capacity in vitro, suggesting that inhibition of the PI3Kδ pathway is the most promising target.

## Results

### Dual inhibition of PI3Kγ and PI3Kδ reduces antitumor immunity of infused CD8^+^ T cells

**Figure 1:**
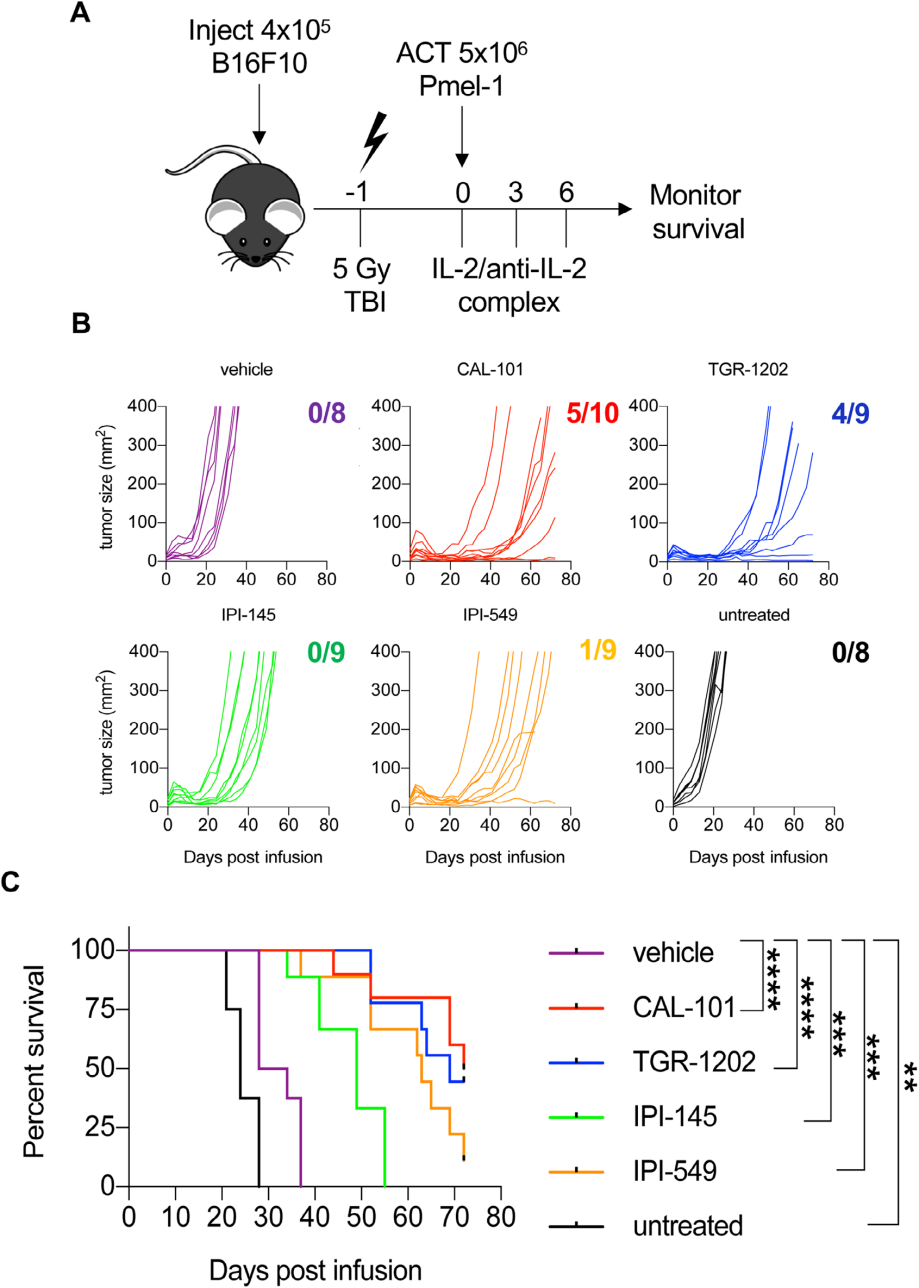
Ex vivo inhibition of PI3Kγ or PI3Kδ promotes robust antitumor immunity against B16F10 melanoma. C57BL/6J mice bearing B16F10 melanoma were treated with 5 × 10^6^ pmel‐1 CD8^+^ T cells expanded for 7 days with vehicle or respective PI3K inhibitors. (A) Experimental schema for ACT. (B) Individual B16F10 tumor growth curves with the number of surviving mice at the end of the study and (C) mouse survival for each group. Data analyzed using Kaplan–Meier survival curves and Log‐rank (Mantel–Cox) test, *n* = 8–10 mice/group from one independent experiment. Statistical significance as follows *p* < 0.05*, *p* < 0.01**, *p* < 0.001***, and *p* < 0.0001****.

To test how blockade of either PI3Kγ and/or PI3Kδ catalytic subunits impacts T cell immunity, peptide‐activated anti‐melanoma pmel‐1 transgenic CD8^+^ T cells were IL‐2 expanded and simultaneously treated with drugs that inhibit either PI3Kγ (IPI‐549), PI3Kδ (CAL‐101 or TGR‐1202), or both PI3Kγ and PI3Kδ subunits (IPI‐145) for 1 week (Note: T cells expanded with 1 μM CAL‐101 were not therapeutic in mice while those expanded with 10 μM CAL‐101 were [35]. Thus, we used this dose (10 μM) for each drug to generate T cell products. Further, as the IC50 for CAL‐101 is 6.5 μM [38], we rationalized that standardizing these various drugs to 10 μM was reasonable). Next, 5 million pmel‐1 cells from these different groups were transferred into lymphodepleted mice bearing B16F10 melanoma (Fig. 1A). Among the drugs tested, we hypothesized that IPI‐145, which coinhibits PI3Kγ and PI3Kδ, would best prevent T cell differentiation ex vivo, thereby leading to improved immunity in vivo. Surprisingly, T cells treated with this dual inhibitor IPI‐145 were the least effective, as the median survival of these mice was approximately 49 days. Conversely, sole blockade of PI3Kγ with IPI‐549 was more effective, as mice infused with these cells lived more than 2 months. Likewise, T cells conditioned with either PI3Kδ inhibitor (TGR‐1202 or CAL‐101) delayed tumor growth and animals survived approximately 70 days (Fig. 1B and C). Vehicle CD8^+^ T cells, as expected, were ineffective, as mice lived for only 1 month post‐ACT and melanoma grew rapidly in untreated mice with a median survival of 24 days. In all instances, T cells treated with any of the inhibitors tested improved survival in mice to greater extent than vehicle‐treated controls (Fig. 1B‐C). However, in contrast to our hypothesis, dual inhibitor IPI‐145 generated the least therapeutic cell product.

### Modulation of the PI3K/AKT/MAPK pathway in T cells

**Figure 2:**
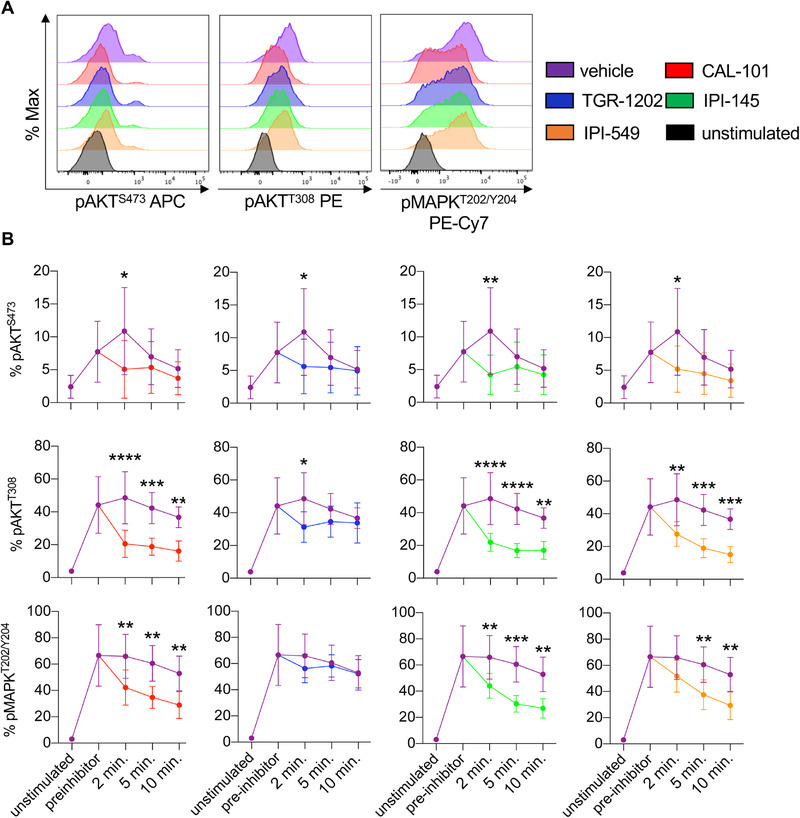
PI3Kγ and/or PI3Kδ inhibitors target the PI3K/AKT/MAPK signaling axis in CD8^+^ T cells. Pmel‐1 splenocytes were stimulated with 1 μM hgp100 for 3 h and then treated with 10 μM PI3K inhibitors. Phosphorylation was analyzed at 2, 5, and 10 min post inhibitor addition. (A) Representative flow cytometry plots of CD8^+^ T cell phosphorylation of AKT^S473^, AKT^T308^, and MAPK^T202/Y204^ 2 min post inhibitor addition. (B) Phosphorylation kinetics of these residues before and after inhibitor addition comparing vehicle to PI3K inhibitor‐treated cells. Data analyzed by unpaired two‐tail *t*‐tests at each time point, *n* = 6 mice/group from two independent studies. All data are represented as the mean ± the SD with statistical significance as *p* < 0.05*, *p* < 0.01**, *p* < 0.001***, and *p* < 0.0001****.

Because T cells treated with dual inhibitor IPI‐145 were less effective at regressing tumors than T cells treated with drugs that only inhibited PI3Kγ or PI3Kδ, we tested if these drugs differentially regulated PI3K activity in T cells. As PI3K is upstream of AKT signaling, phosphorylation of AKT^S473^ and AKT^T308^ were measured in pmel‐1 CD8^+^ T cells over time [39]. All inhibitors reduced the pAKT^S473^ and pAKT^T308^ in pmel‐1 within 2 min of treatment compared to vehicle controls (Fig. 2A and B). Within 10 min, pAKT^T308^ rebounded in pmel‐1 treated with TGR‐1202 to the levels seen in vehicle cells. Conversely, pAKT^T308^ remained significantly lower in pmel‐1 conditioned with IPI‐145, CAL‐101, and IPI‐549 compared to vehicle controls (Fig. 2B). Other signaling events induced by TCR activation, such as MAPK, mTOR, and STAT5, were tested. While pMAPK^T202/Y204^ was reduced in inhibitor‐treated T cells, phosphorylated S6 protein^S235/236^, a measure of mTOR activity as well as STAT5^Y694^ were not altered (Supporting Information Fig. 1 and 2). Our data reveal that all the inhibitors transiently blocked components of the PI3K signaling axis in T cells but did not mediate off‐target effects on other pathways including mTOR or STAT5.

### PI3K blockade slows CD8^+^ T cell differentiation and proliferation to preserve memory formation

**Figure 3:**
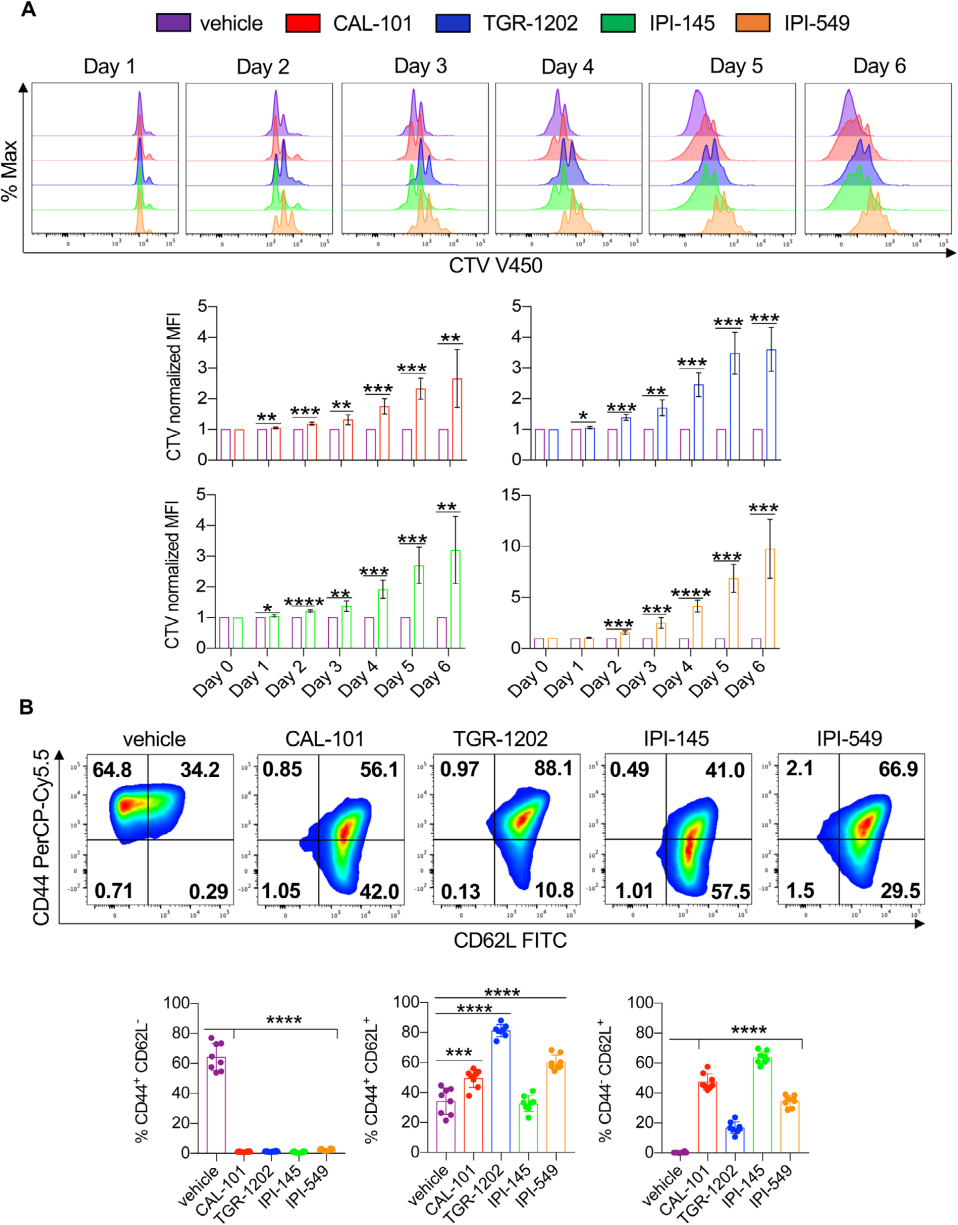
PI3K inhibitors reduce the differentiation and proliferation of CD8^+^ T cells. Pmel‐1 were stimulated with 1 μM hgp100 and cultured in the presence or absence of PI3K inhibitors and IL‐2 for 1 week. (A) Representative flow cytometry plots (top) and quantification (bottom) of CD8^+^ T cells stained with cell trace violet from Day 0 to 6. Data analyzed by one‐sample t‐test at each time point, *n* = 6 mice/group from three independent experiments. (B) Representative flow cytometry plots (top) and expression (bottom) of CD8^+^ memory populations (Day 7). Data analyzed by one‐way ANOVA with Tukey's multiple comparisons, *n* = 8 mice/group from two independent experiments. All bars represent the mean ± the SD with statistical significance as *p* < 0.05*, *p* < 0.01**, *p* < 0.001***, and *p* < 0.0001****.

**Figure 4:**
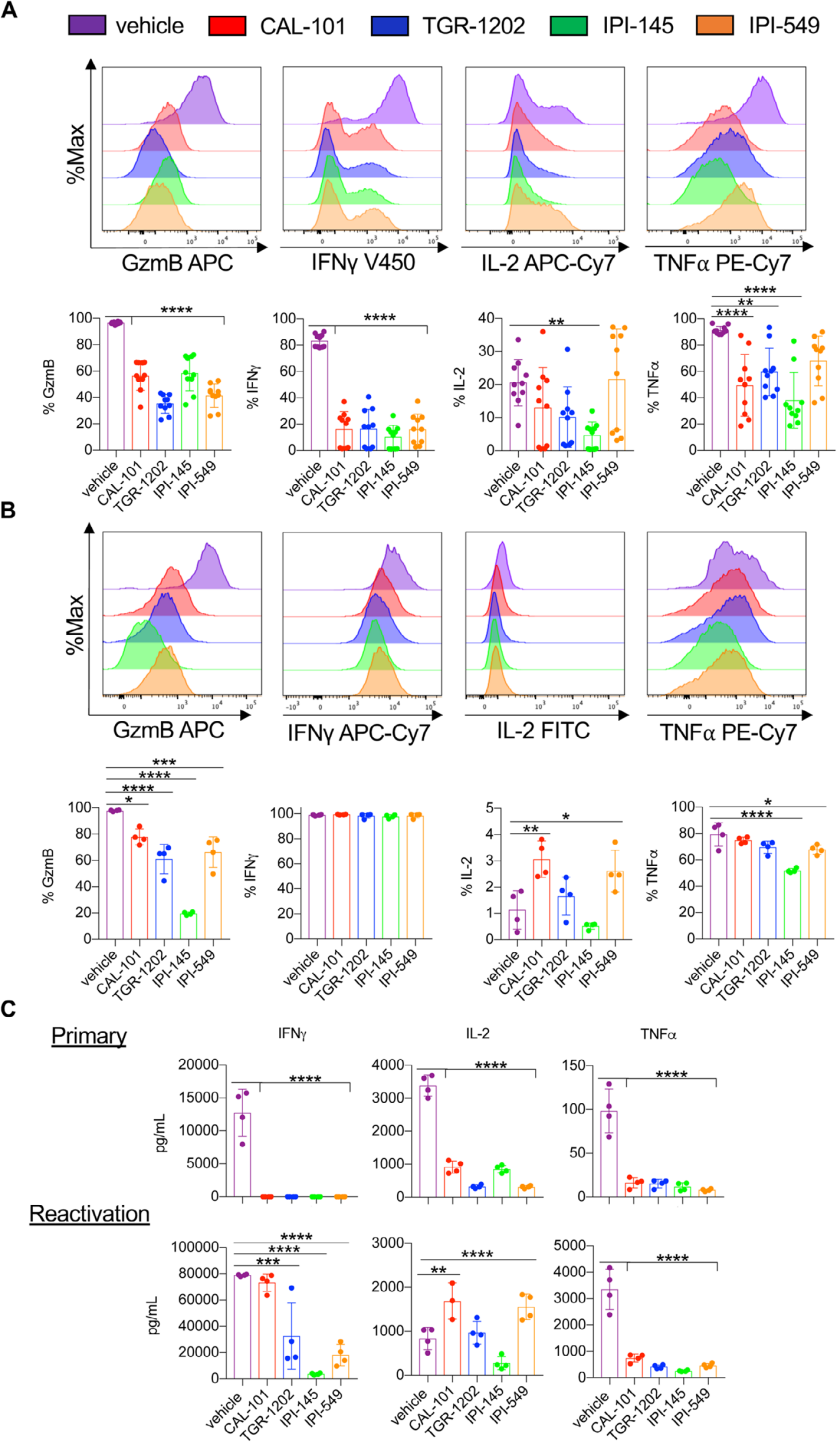
Functional rebound to tumors is compromised in IPI‐145 treated pmel‐1 T cells. Representative flow cytometry plots and expression of intracellular cytokines from primary activation (A) and reactivated pmel‐1 (secondary) (B). Note for primary stimulation, pmel‐1 were cultured for 8 days and stimulated with irradiated splenocytes loaded with 1 μM hgp100 in inhibitor‐free media for 4 h (*n* = 10 mice/group from three independent experiments). For secondary re‐stimulation cytokine staining, Day 7 CD8^+^ pmel‐1 cells were stimulated with irradiated splenocytes loaded with 1 μM hgp100 in inhibitor‐free media and grown for an additional 3 days. Cells were stimulated on Day 10 with irradiated splenocytes loaded with 1 μM hgp100 in inhibitor‐free media for 4 h (*n* = 4 mice/group from one independent experiment). (C) IFNγ, IL‐2, and TNFα production from primary (Day 2) and secondary (Day 10) stimulated pmel‐1 (the latter without inhibitor) measured by ELISA, *n* = 3–4 mice/group from one independent experiment. Data analyzed by one‐way ANOVA with Tukey's multiple comparisons. All bars represent the mean ± the SD with statistical significance as *p* < 0.05*, *p* < 0.01**, *p* < 0.001***, and *p* < 0.0001****.

We assumed that PI3K inhibitor treatment would slow the expansion of T cells. Indeed, we found that all inhibitors delayed T cell proliferation compared to untreated vehicle T cells, indicated by their slowed dilution of cell trace violet dye (CTV) over time (Fig. 3A, Supporting Information Fig. 3, Fig. 4A). The PI3Kγ inhibitor (IPI‐549) stalled T cell proliferation the most (Fig. 3A). However, ample numbers of viable CD8^+^ T cells could be expanded for ACT therapy (Supporting Information Fig. 4B).

**Figure 5:**
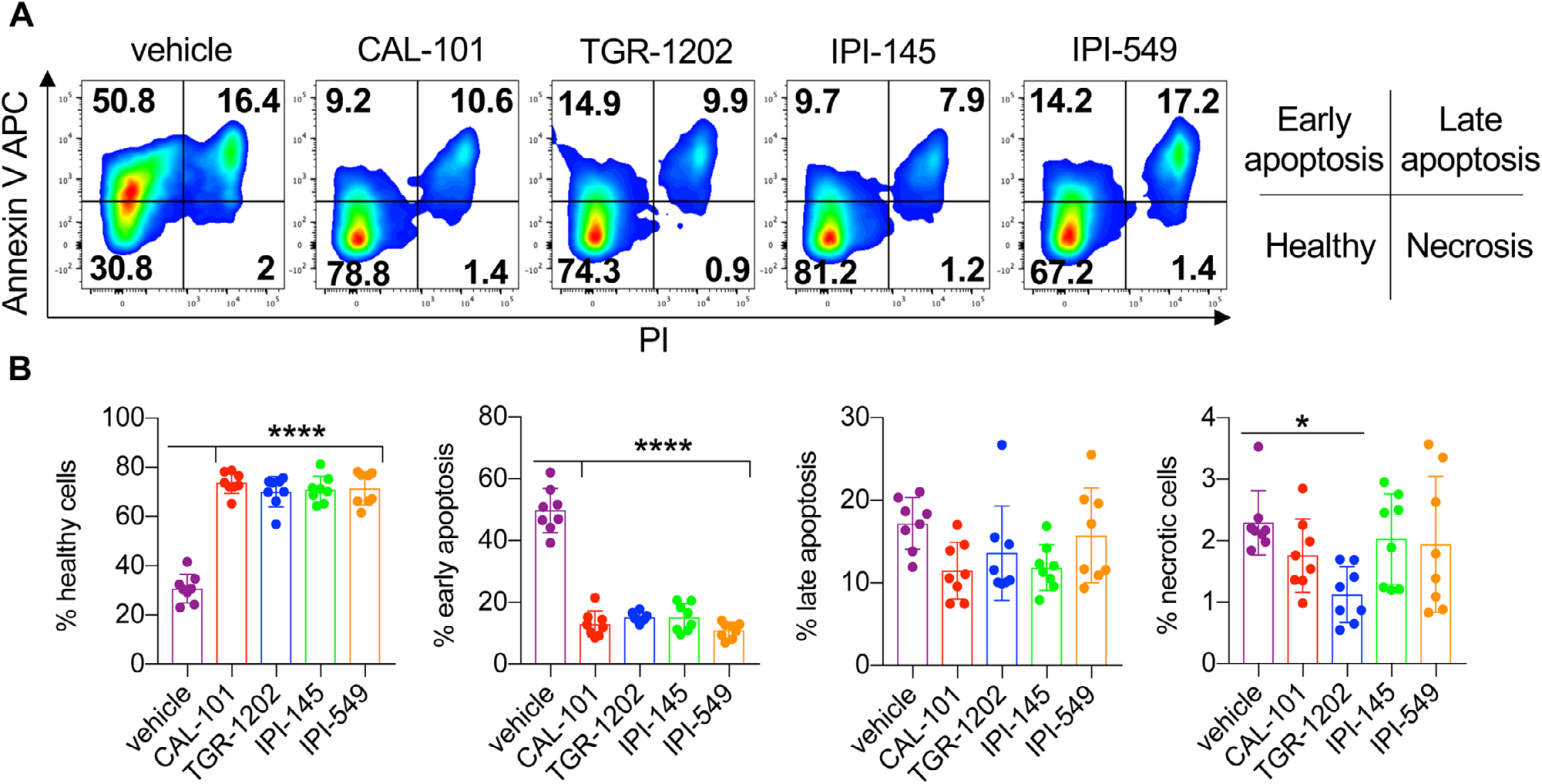
Inhibitor‐treated CD8^+^ T cells are resistant to activation‐induced cell death. Day 7 CD8^+^ pmel‐1 cells were stimulated overnight with plate bound anti‐CD3 in inhibitor‐free media. (A) Representative flow cytometry plots of CD8^+^ T cells and (B) proportions of cells in apoptotic stages as defined by Annexin V and PI staining. Data analyzed by one‐way ANOVA with Tukey's multiple comparisons with *n* = 8 mice/group from two independent experiments. All bars represent the mean ± the SD with statistical significance as *p* < 0.05*, *p* < 0.01**, *p* < 0.001***, and *p* < 0.0001****.

**Figure 6:**
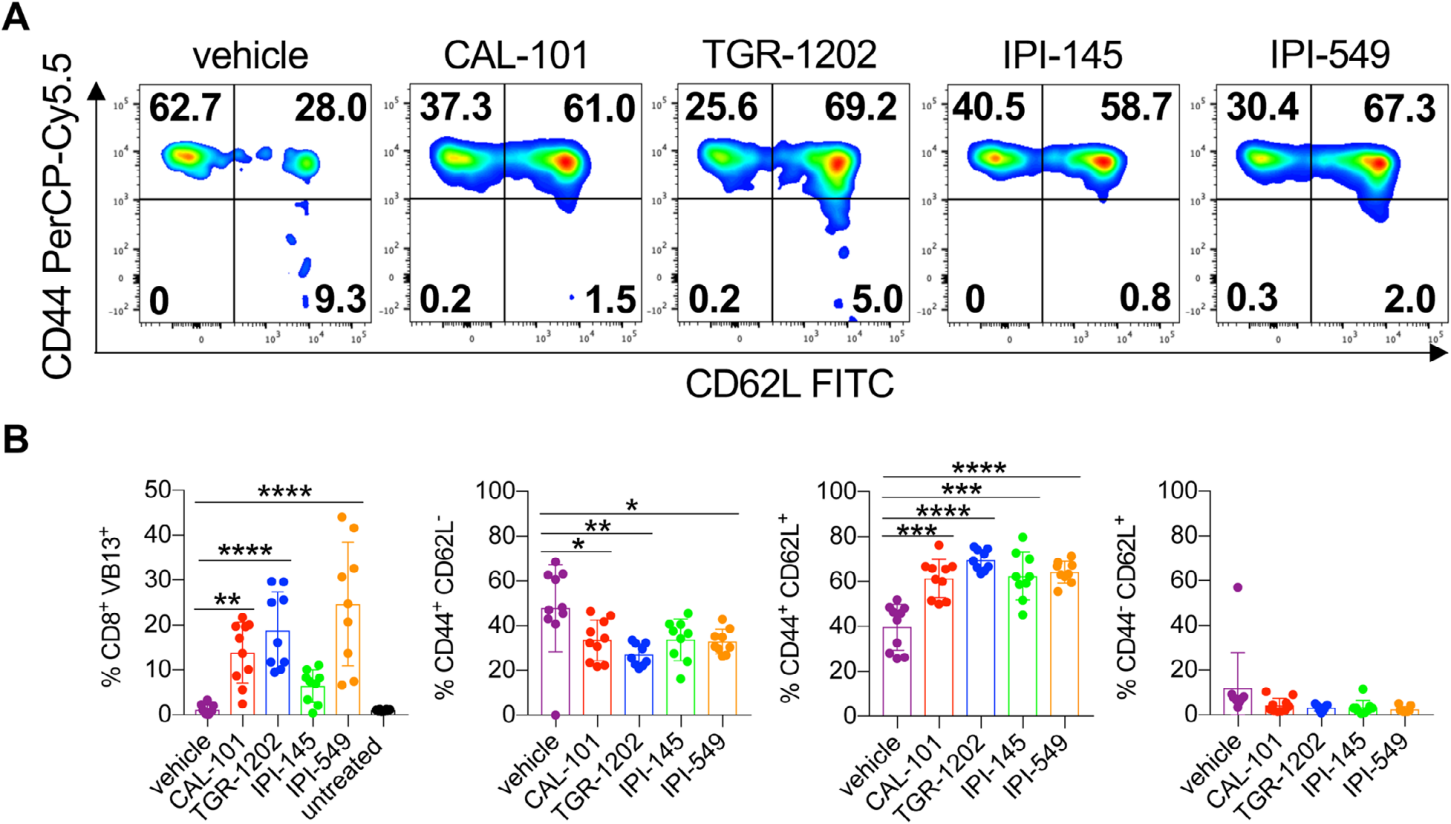
Decreased in vivo persistence of CD8^+^ T cells treated with IPI‐145. Day 14 analysis of donor CD8^+^ T cells from the peripheral blood of C57BL/6J melanoma bearing mice with representative memory profile flow cytometry plots (A) and donor cell persistence and memory phenotype (B). Data analyzed by one‐way ANOVA with Tukey's multiple comparisons, *n* = 8–10 mice/group from one independent experiment. All bars represent the mean ± the SD with statistical significance as *p* < 0.05*, *p* < 0.01**, *p* < 0.001***, and *p* < 0.0001****.

T cells differentiated to a full‐effector memory state are less therapeutic than younger less differentiated cohorts when infused into tumor bearing hosts [12]. Thus, we assessed the phenotype of inhibitor‐treated versus vehicle T cells. As reported, 1 week after expansion, most vehicle pmel‐1 CD8^+^ T cells possessed an effector memory phenotype (CD44^+^CD62L^−^) while inhibitor‐treated pmel‐1 had a mainly central (CD44^+^CD62L^+^) or naive/stem‐like (CD44^−^CD62L^+^) memory profile (Fig. 3B). Nearly half the pmel‐1 possessed a naïve/stem memory‐like profile when expanded with CAL‐101 or with IPI‐145. Conversely, a majority of the pmel‐1 expanded with TGR‐1202 or IPI‐549 possessed a central memory phenotype (Fig. 3B). Other markers associated with memory, Ly6C and CD103, were also differentially expressed on vehicle versus inhibitor‐treated T cells (Supporting Information Fig. 4C, Fig. 5). Note, T cell activation was not impaired by these drugs, as CD69 was induced 1 day after peptide stimulation (Supporting Information Fig. 6A). Yet, 5 days after activation, inhibitor‐treated T cells downregulate CD69 while vehicle T cells maintained expression (Supporting information Fig. 4C, Fig. 6A). Additional investigation revealed that, in order to generate naïve/stem‐like T cells with CAL‐101 and IPI‐145, these drugs needed to be added 3 h after peptide activation, as later in hibitior addition (24 h after peptide activation) T cells possessed a central memory phenotype (Supporting information Fig. 6B). These data are not surprising given reports showing that T cell priming requires approximately 20 h to manifest after antigen recognition [40, 41]. Our results reveal that blocking PI3K signaling early, during priming, more effectively reduces T cell differentiation, in turn generating a greater frequency of antitumor T cells with naïve/stem‐like memory profile.

### PI3K inhibition reduces T cell exhaustion under chronic antigen stimulation

As blocking PI3K signaling in T cells prevents effector/terminal differentiation, we next tested our idea that these “younger” inhibitor‐treated cells would be resistant to exhaustion in the tumor. To model exhaustion, T cells were chronically stimulated with melanoma antigen every day for 3 days and then assayed for their viability the subsequent day (Supporting information Fig. 7A). Indeed, most vehicle T cells were exhausted, as most died in this assay due to chronic antigenic insult. Remarkably, half of the inhibitor‐treated T cells remained viable (Supporting information Fig. 7B). However, PI3K inhibition did not prevent T cell activation, as inhibitor‐treated T cells expressed CD69 in the presence of antigen. Yet these cells resisted transitioning to a terminally differentiated state, evidenced by their low expression of Klrg1 and Ly6C compared to vehicle cohorts (Supporting information Fig. 7C). Conversely, the few vehicle cells that did survive were effector memory while all PI3K‐inhibited T cells maintained a central memory phenotype (Supporting information Fig. 7D). Our results indicate that PI3K blockade protects T cells from activation‐induced exhaustion by tumor antigen while preserving their survival and memory phenotype.

### Dual PI3Kγ and PI3Kδ blockade impairs CD8^+^ T cell function

We next tested how these inhibitors impacted T cell cytokine production after primary and secondary stimulation with tumor antigen. After primary expansion, vehicle T cells secreted more granzyme B, IFNγ, IL‐2, and TNFα than those conditioned with any of the PI3K inhibitors tested (Fig. 4A, Supporting information Fig. 8). However, T cells initially treated with inhibitors that solely blocked PI3Kγ or PI3Kδ could rebound functionally when reactivated with tumor antigen evidenced by their regained capacity to secrete cytokines, albeit granzyme B and TNFα were produced to a lesser extent than reactivated vehicle cohorts (Fig. 4B). Dual‐inhibited T cells were the most compromised in their capacity to secrete granzyme B, IL‐2 and TNF, even after cells were reactivated with antigen.

To confirm this observation, cytokines from the supernatant of pmel‐1 cultures were measured during primary stimulation (Day 2) and after their reactivation (Day 10). PI3K inhibition during primary stimulation suppressed IFNγ, IL‐2, and TNFα production (Fig. 4C). However, IFNγ production by inhibitor‐treated T cells rebounded upon restimulation. Similar to our intracellular cytokine data, T cells treated with the dual inhibitor (IPI‐145) were compromised in their capacity to functionally rebound, evidenced by the reduced secretion of IFNγ (Fig. 4C). T cells treated with CAL‐101 and IPI‐549 secreted more IL‐2 while TNFα production was compromised in all inhibitor‐treated T cells compared to the vehicle cohort (Fig. 4C). Overall, our results indicate that dual blockade of PI3Kγ and PI3Kδ impairs CD8^+^ T cell recall, as they were unable to fully rebound functionally after re‐encountering antigen, perhaps explaining why they were less effective in vivo.

### Ex vivo PI3Kγ and PI3Kδ inhibition reduces T cell in vivo persistence

As dual blockade of PI3Kγ and PI3Kδ impaired CD8^+^ T cell function ex vivo, we posited that their viability may also be hampered. Post anti‐CD3 reactivation, we found that most vehicle T cells were apoptotic, undergoing both early and late stages of apoptosis (Fig. 5A and B). In contrast, T cells treated with the PI3K inhibitors were healthy, indicated by their minimal induction of apoptosis (Fig. 5A and B). Likewise, PI3K‐inhibited T cells had improved persistence in vivo compared to the vehicle cohort (Fig. 6A‐B). Transferred T cells that were conditioned ex vivo with single PI3K subunit inhibition persisted best, which directly correlated with improved treatment outcome (Fig. 1A‐C, 6A and B). Moreover, the majority of PI3K‐inhibited T cells in the blood of mice possessed a central memory phenotype (CD44^+^CD62L^+^) compared to vehicle cells (Fig. 6A and B). Thus, our data suggest that blockade of a single PI3K subunit is ideal for generating potent T cells for clinical translation.

### PI3Kδ inhibition improves cytotoxicity of Meso‐CAR T cells

**Figure 7:**
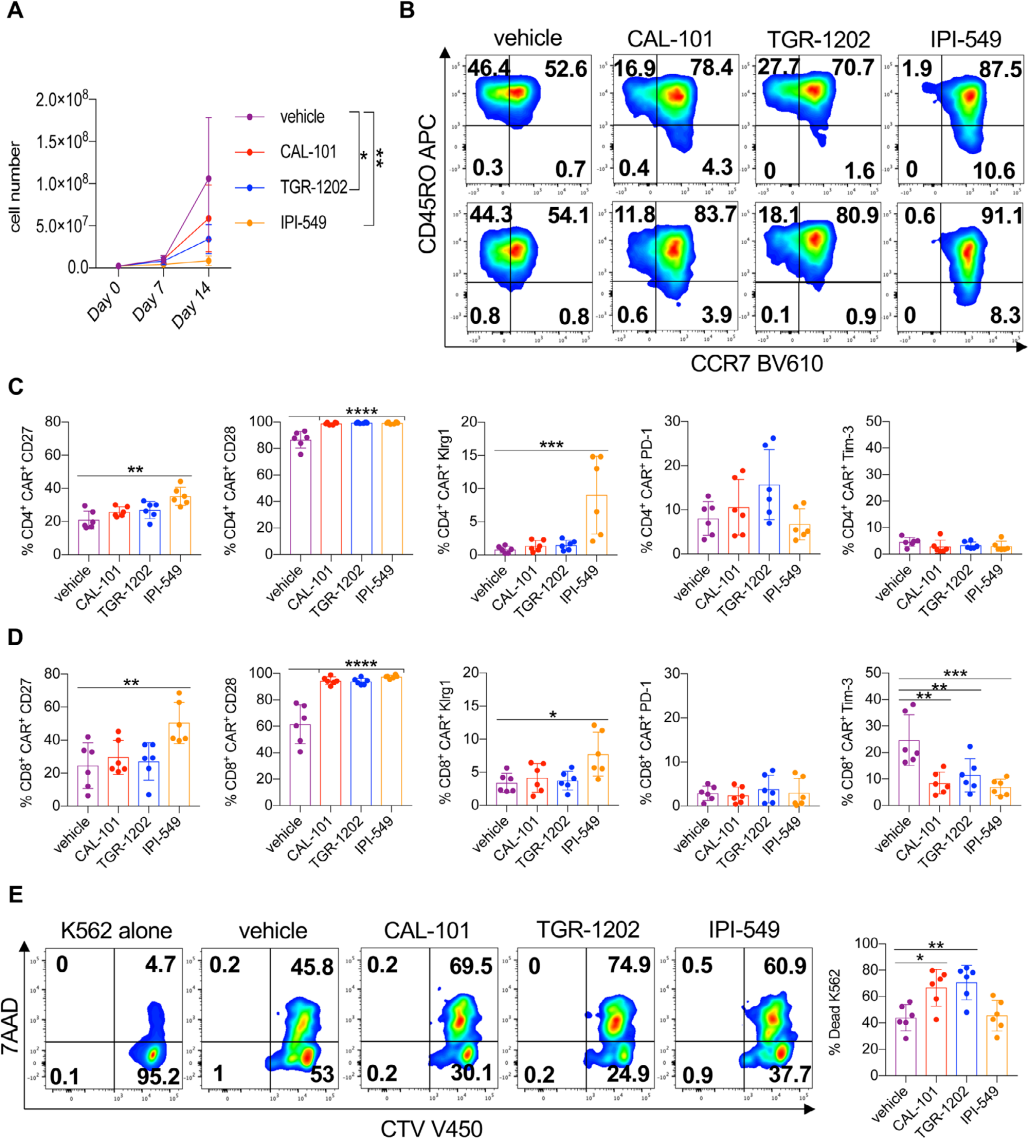
PI3Kδ reduces differentiation of human Meso‐CAR T cells while preserving cytolytic capacity. Healthy donor T cells were stimulated with anti‐CD3/CD28 beads and transduced with a mesothelin‐specific 4‐1BBζ CAR in the presence or absence of PI3K inhibitors. (A) Expansion of vehicle and inhibitor‐treated CAR T cell cultures over 2 weeks. (B) Representative flow cytometry plots of CD4^+^ CAR^+^ (top) and CD8^+^ CAR^+^ (bottom) T cell memory profiles on Day 7 of expansion. Expression of costimulatory and inhibitory receptors of CD4^+^ CAR^+^ (C) and CD8^+^ CAR^+^ (D) T cells on Day 7 of expansion. (E) Representative flow cytometry plots and quantification of K562‐meso cell death with total T cells in inhibitor‐free media on Day 15. Cell growth analyzed by one‐way ANOVA with Tukey's multiple comparisons at each time point. Surface expression and cytotoxicity data analyzed by one‐way ANOVA with Tukey's multiple comparisons, *n* = 6 healthy donors/group from two independent experiments. All bars represent the mean ± the SD with statistical significance as *p* < 0.05*, *p* < 0.01**, *p* < 0.001***, and *p* < 0.0001****.

CAR T cell therapy is effective in some patients with hematological malignancies but less promising in individuals with solid tumors. To improve this therapy, we tested if PI3K inhibitor treatment could augment CAR T cell phenotype and cytotoxicity [4, 42]. Thus, peripheral blood T cells from human healthy donors were activated with anti‐CD3/CD28 beads, transduced with a mesothelin‐specific CAR containing 4‐1BB and CD3ζ signaling domains and expanded with IL‐2 in the presence of PI3Kγ or PI3Kδ inhibitors for 2 weeks. PI3Kδ inhibitors slightly reduced T cell yield while IPI‐549 suppressed T cell expansion more than TGR‐1202 or CAL‐101 (Fig. 7A).

PI3K inhibitors slowed the differentiation of both CD4^+^ and CD8^+^ CAR T cells, marked by elevated CCR7 expression, with IPI‐549 treatment best supporting this phenotype (Fig. 7B, Supporting information Fig. 9). Likewise, CD27 was elevated on T cells treated with IPI‐549, whereas T cells treated with any PI3K inhibitor expressed more CD28 (Fig. 7C and D). PD‐1 and Tim‐3 were similarly expressed on CD4^+^ CAR T cells while Tim‐3 was reduced on PI3K‐inhibited CD8^+^ CAR T cells (Fig. 7C and D). IPI‐549‐treated T cells expressed more Klrg1 than vehicle, CAL‐101 and TGR‐1202 cohorts. Yet, in our in vitro cytotoxic assay (Supporting information Fig. 10), we found that IPI‐549‐treated and vehicle CAR T cells killed approximately 50% of the mesothelin‐expressing cancer cells while CAL‐101‐ and TGR‐1202‐treated T cells killed more (∼75%) of them (Fig. 7E). Our findings indicate that PI3Kδ blockade generates human CAR T cells with potent cytolytic activity.

## Discussion

Dogma states that many T cells are needed to effectively treat patients with ACT therapy [43, 44]. Yet, preclinical work is now debunking this idea, revealing that fewer T cells with a less differentiated state can mount robust responses against tumors [8, 12, 13, 15, 17, 35, 45]. Thus, research efforts have focused on divorcing T cell expansion from differentiation [8, 12, 46]. Previous work by our group has shown that PI3Kδ inhibition with CAL‐101 generates T cells with naïve/stem memory‐like properties, thereby improving their therapeutic efficacy [35, 36]. Herein, we report for the first time that ex vivo inhibition of PI3Kγ activity with IPI‐549 endows murine T cells with similar therapeutic efficacy when infused into mice compared to those conditioned with PI3Kδ inhibitors. Simultaneous ex vivo inhibition of PI3Kγ and PI3Kδ further blunts T cell differentiation, best preserving their naïve/stem memory phenotype. Yet, these cells are surprisingly ineffective compared to T cells inhibited solely of PI3Kγ or PI3Kδ. Our work implies other factors beyond T cell “youth” are involved in generating therapeutic ACT products for patients.

Beyond phenotype, engraftment of infused T cell products is another determinant of successful ACT therapy [47, 48, 49]. The survival benefit endowed by PI3K inhibition is intriguing, as their capacity to prevent apoptosis upon antigenic re‐encounter may contribute to their increased efficacy. However, dual inhibition of PI3Kγ and PI3Kδ seemed deleterious compared to single subunit PI3K inhibition, indicated by their reduced ability to rebound functionally and persist, all may explain their reduced ability to clear tumors. As PI3Kγ and PI3Kδ are influenced by TCR signaling and induce T cell activation, functional redundancies of these subunits likely exist. This redundancy may ensure function upon secondary antigen encounter if either PI3Kγ or PI3Kδ is inhibited but lost when both are blocked.

With murine and human lymphocytes, we found that inhibition of PI3Kγ or PI3Kδ generated T cell products with a less differentiated memory phenotype. However, in human CAR T cells, PI3Kγ inhibition limited T cell expansion and increased Klrg1, suggesting this inhibitor promoted premature senescence [50, 51]. Indeed, Klrg1 has been associated with reduced T cell function. Moreover, coinhibition of Klrg1 and PD‐1 improves immunity to melanoma, colorectal, and breast cancer [52, 53]. Thus, our new body of work might imply that ex vivo inhibition of both Klrg1 and PI3Kγ could improve antitumor immunity.

One question that remains unclear in our study is exactly how ex vivo PI3K inhibition bolsters the antitumor activity of T cells. Do these inhibitors have a direct effect on the T cells in the culture? Or do the inhibitors indirectly augment T cells by modulating other immune cells transiently present at the start of the culture? For example, PI3Kδ signaling is essential for Tregs and mice deficient of PI3Kδ signaling have impaired Treg survival, which in turn unleashes the cytolytic function of CD8^+^ T cells against the tumors [18, 22, 38]. Likewise, PI3Kγ alters the inflammatory/anti‐inflammatory balance in macrophages [34], which may explain why our T cell cultures better regress tumors. Future studies will be important to disentangle these questions.

Our work clearly shows that not all PI3K inhibitors are created equal and each inhibitor bestows T cell cultures with different properties. Ultimately, inhibition of a single PI3K subunit is sufficient to propagate T cells with robust potency, and this may be the result of conditioning that allows them to functionally rebound and persist in vivo. Additionally, PI3Kγ inhibition mediated differential effects on mouse versus human T cells in our studies with the dose used to generate them. Our findings have immediate implications supporting that PI3Kδ is the best therapeutic target in the PI3K/AKT pathway, and drugs that block it may improve the efficacy of ACT products to treat patients with large, established solid tumors.

## Materials and methods

### Mice and cell lines

C57BL/6J and pmel‐1 TCR‐transgenic (B6.Cg‐Thy1^a^/Cy Tg(TcraTcrb)8Rest/J) mice were obtained from Jackson Laboratories and housed and bred in the Hollings Cancer Center at the Medical University of South Carolina. The B16F10 (H‐2b) melanoma cell line used for ACT therapy experiments was a gift from the Nicholas Restifo lab at the NCI. The K562‐mesothelin expressing cell line used in the in vitro CAR cytotoxicity assay was a gift from the Carl June lab at the University of Pennsylvania. Both cell lines were expanded in cell culture media (RPMI 1640 containing L‐glutamine, 10% FBS, 1% Pen/Strep, nonessential amino acids, sodium pyruvate, and 0.1% BME and HEPES).

### Mouse and human T cell culture

Splenocytes were collected from both male and female pmel‐1 TCR‐transgenic mice ages 8 to 14 weeks and stimulated with 1 μM hgp100 peptide (GenScript) in 24 well plates. Three hours post‐TCR stimulation, splenocytes were treated with 200 IU rhIL‐2 (NCI) and either vehicle (DMSO) or 10 μM CAL‐101, TGR‐1202, IPI‐145, or IPI‐549 (Selleckchem). For experiments analyzing the effects of PI3K inhibition during T cell priming, PI3K inhibitors were added 24 h post‐TCR stimulation. During ex vivo expansion, cells were given 200 IU rhIL‐2 and media containing vehicle or respective inhibitor on Days 3–6.

Human donor peripheral blood was obtained from the Sylvan N. Goldman Oklahoma Blood institute. Pan‐T cell isolation was conducted using a negative bead selection protocol (Invitrogen). T cells were stimulated at a 1:1 ratio with anti‐CD3/CD28 beads (Gibco) with 200 IU rhIL‐2 and either vehicle or 10 μM of respective inhibitor. Day 2 postactivation, T cells were magnetically debeaded and transduced with a lentivirus containing a mesothelin‐specific CAR containing CD3ζ and 4‐1BB signaling domains (Gift, Carl June) [54]. Cells were expanded for a total of 14 days in the presence of vehicle or 10 μM of respective inhibitor and 200 IU rhIL‐2 was added daily.

### ACT therapy

B16F10 melanoma cells were thawed from cryopreservation, resuspended in culturing media in a T175 flask, and expanded for 1 week. Four days prior to ACT, 4 × 10^5^ B16F10 melanoma cells were injected subcutaneously onto the abdomen of C57BL/6J mice. C57BL/6J mice were all 8‐week‐old females and were randomized prior to ACT to standardize tumor growth. B16F10‐bearing mice received nonlethal total body irradiation (5 GY) 1 day prior to ACT. Pmel‐1 CD8^+^ T cells were stimulated with irradiated splenocytes loaded with 1 μM hgp100 peptide at a 1 irradiated splenocyte:10 T cell ratio overnight in the presence of vehicle or 10 μM of PI3K inhibitor 1 day prior to ACT. Five million CD8^+^ T cells were transferred to each mouse via tail vein injection, and mice received three intraperitoneal injections of IL‐2/anti‐IL‐2 complex (1.5 μg rhIL‐2 (NIH) and 7.5 μg IL‐2 Mab clone 5355 [R&D Systems]) to boost donor cell engraftment on Days 0, 3, and 6 postinfusion [55]. Tumors were measured twice weekly with calipers in a blinded manner and donor cell persistence was measured by flow cytometry of the peripheral blood obtained from the submandibular vein on Day 14 postinfusion.

### Flow cytometry analysis

Cultured cells were collected and counted using a Countess automated cell counter (Invitrogen) and 2–4 × 10^5^ cells were plated and washed with FACS wash buffer (PBS with 2% FBS). Cells were stained for 20 min at 4°C with the following monoclonal antibodies. Mouse antibodies: CD8 (53‐6.7), vβ13 (MR12‐3), CD25 (PC61), CD103 (M290), IL‐2 (JES6‐5H4), Klrg1 (2F1), and streptavidin were purchased from BD Bioscience. CD44 (IM7), CD62L (MEL‐14), CD69 (H1.2F3), Ly6C (HK1.4), granzyme B (GB11), IFNγ (XMG1.2), and TNFα (MP6‐XT22) were purchased from Biolegend. To measure phosphorylation of proteins relevant to PI3K‐mediated signal transduction, pmel‐1 splenocytes were stimulated ex vivo with 1 μM hgp100 for 3 h at 37°C. Following TCR stimulation, vehicle or 10 μM of the respective inhibitor were added to each well. At 2, 5, and 10 min post inhibitor addition, cells were fixed with 16% (1.6% final) paraformaldehyde for 10 min, were washed and treated with 100% methanol for 30 min on ice. Following fixation, cells were stained with pAKT^S473^ and pSTAT5^Y694^ (BD Bioscience) and pAKT^T308^, pMAPK^T202/Y204^, and pS6 protein^S235/236^ (Cell Signaling Technology) for 1 h at room temperature.

Monoclonal antibodies used to stain human T cells were CD45RO (UCHL1), CCR7 (G043H7), CD28 (CD28.2), Klrg1 (SA231A2), PD‐1 (EH12.2H7), Tim‐3 (F38‐2E2), CD4 (OKT4), and CD8 (RPA‐T8) purchased from Biolegend. CD27 (M‐T271) was purchased from BD Bioscience. To identify CAR^+^ T cells, human T cells were stained with polyclonal antibody anti‐mouse IgG F(Ab’)2 from Jackson ImmunoResearch for 20 min at room temperature followed by the remaining surface antibodies for 20 min. To identify viable cells, fixable zombie aqua viability dye (Biolegend) was used. Flow cytometry samples were analyzed on the BD FACSVerse or the BD Fortessa X‐20 instruments at the Medical University of South Carolina. Data were analyzed using FlowJo software v10 (BD).

### Functional assays

For the CTV proliferation experiments, 4 × 10^6^ splenocytes were stained with 2.5 μM CTV (Invitrogen) in PBS at 37°C for 30 min. To dilute the CTV, 1 mL of FBS was added to the mixture and incubated at room temperature for 10 min. Cells were pelleted, resuspended in cell culture media, and plated 1 × 10^5^ cells/well in a 96‐well round bottom plate. Cell proliferation was assessed by flow cytometry on Days 0–6. To measure apoptosis, 96‐flat bottom wells were coated with 1 μg/mL of purified anti‐CD3 (145‐2C11) (Biolegend) for 12 h at 4°C. Inhibitor‐treated or vehicle CD8^+^ T cells were washed, plated in inhibitor‐free media, and incubated at 37°C for 12 h. The following day, cells were stained using the propidium iodide annexin V kit (Biolegend). For the chronic stimulation assays, T cells were collected on Day 7 and 4 × 10^5^ pmel‐1 CD8^+^ T cells stimulated 1:1 with irradiated splenocytes loaded with hgp100 in the presence of vehicle or 10 μM of respective inhibitor. On Days 8 and 9, 200 μL of media was removed from each well, T cells were stimulated with 2 × 10^5^ irradiated splenocytes in 200 μL of media containing vehicle or 10 μM inhibitor and analyzed by flow cytometry on Day 10. Cytotoxic potential of the human CAR T cells was measured by staining K562‐Meso cells with CTV (Invitrogen) for 20 min in the dark at room temperature. K562‐Meso cells were then mixed with cultured human T cells at 2 × 10^3^ K562‐Meso cells to 1 × 10^4^ T cells and incubated at 37°C for 12 h. For this assay, CAR T cells and K562 target cells were in inhibitor‐free media. 7AAD (BD Pharmigen), CD4, and CD8 surface antibodies were stained after incubation.

Intracellular cytokines were measured by stimulating 1 × 10^5^ CD8^+^ T cells on Day 8 with irradiated splenocytes loaded with 1 μM hgp100 peptide at a 1:1 ratio for 4 h in inhibitor‐free media. Brefeldin A (1 μg) and Monensin (2 μM) were added to each well upon stimulation. To measure cytokine production upon restimulation, cells were placed in fresh inhibitor‐free media on Day 7 and 2 × 10^6^ CD8^+^ pmel‐1 cells were restimulated with 2 × 10^5^ irradiated splenocytes loaded with 1 μM hgp100. On Day 10, 1 × 10^5^ CD8^+^ T cells were stimulated at a 1:1 ratio with irradiated splenocytes loaded with 1 μM hgp100 for 4 h. The intracellular cytokine staining protocol and fixation kit were used from Biolegend. Supernatant cytokine analysis was conducted by Eve Technologies using their mouse cytokine array/chemokine 32‐plex array. Supernatant was collected from cell cultures during primary stimulation (Day 2) and after restimulation (Day 10).

### Statistics

Mouse survival for ACT therapy experiments was analyzed using Kaplan–Meier survival curves and the Log‐rank (Mantel–Cox) test. Comparisons between PI3K inhibitors for T cell surface phenotype, intracellular cytokine production, apoptosis, in vivo persistence, and CAR cytotoxicity were analyzed using the one‐way ANOVA test with Tukey's multiple comparisons. Unpaired two‐tail T tests were used to analyze phosphorylation of signal transducers at each timepoint after inhibitor addition. CTV fluorescence was analyzed with one‐sample *t*‐tests at each time point. One‐way ANOVA with Tukey's multiple comparisons was used for human CAR T cell expansion at each time point. Statistical analysis was conducted using GraphPad prism 7 software (GraphPad). All bars represent the mean ± SD with statistical significance as *p* < 0.05*, *p *< 0.01**, *p* < 0.001***, and *p* < 0.0001****.

### Study approval

Mouse studies were approved by the IACUC of the Medical University of South Carolina Animal Resource Center (ARC#3039). The MUSC Institutional Review Board approved the work with healthy human donor PBMCs (pro 13570) and the Institutional Biosafety Committee approved the use of virus to generate CAR T cells (IBC 381).

## Funding

This research was supported by NIH T32AI132164‐01 and NIH T32DE017551 (CJD); Abney Foundation Scholarship (DCA); NIH T32GM008716‐19 (GORR); NCI F30CA243307, NIH T32GM008716, and NIH T32DE017551 (HMK), Hollings Cancer Center Graduate Fellowship (ASS); NIH R50CA233186 (MMW); NIDCR K08DE026542 (DMN), NIH R01CA222817, PO1CA154778 and the Cancer Research Institute (MPR); NIH R01CA175061, R01CA208514 (CMP), and P30 CA138313 to the Cell Evaluation & Therapy Shared Resource, Hollings Cancer Center, Medical University of South Carolina.

## Author Contributions

CJD and CMP were responsible for the design and conception of the study. CJD and DCA conducted experiments and CJD performed statistical analysis of the data. CJD and GORR designed and created figures for the manuscript. CJD and CMP wrote the manuscript and all the others provided edits for the manuscript.

## Conflict of interest

CMP has a provisional patent on pharmaceutical drug combinations or genetic strategies that instill durable antitumor T cell memory and activity (patent application P1685).

**Abbreviations**
ACT: adoptive T cell transfer
CAR: chimeric antigen receptor
CTV: cell trace violet
PI3K: phosphoinositide 3‐kinase

## Supporting information

Supporting Information.
