# Supplementary material for: Ex vivo blockade of PI3K gamma or delta signaling enhances the antitumor potency of adoptively transferred CD8+ T cells
Source: Eur J Immunol. 2020 May 28;50(9):1386–99. doi: 10.1002/eji.201948455 (PMC7496332; doi:10.1002/eji.201948455)
Supplement: Supplementary file 1 — Supporting Information. [file EJI-50-1386-s001.pdf]

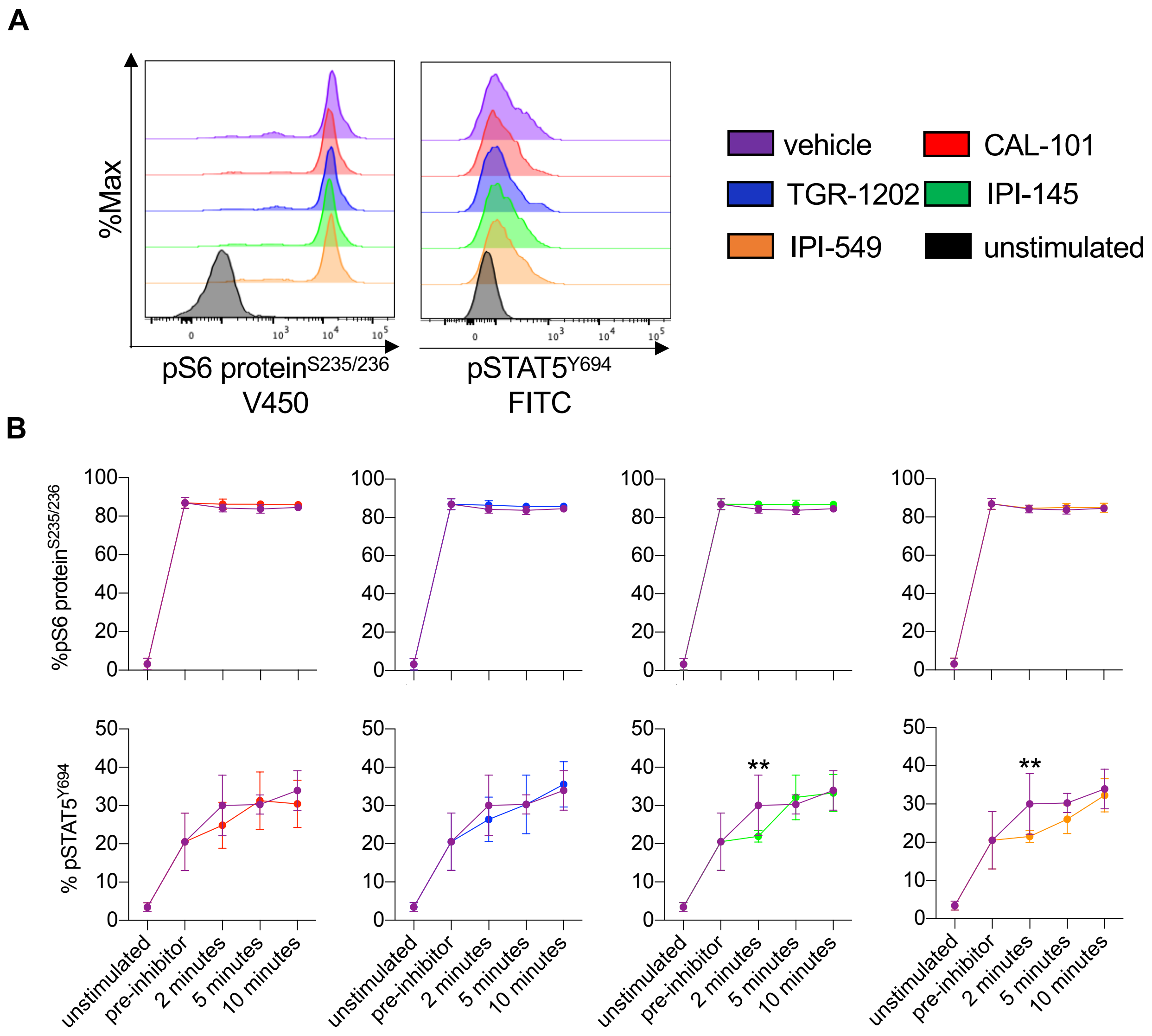

Figure S1: PI3Kγ and/or PI3Kδ inhibition does not impair STAT5 or mTOR signaling in $CD8^+$ T cells. Pmel-1 splenocytes were stimulated with 1μM hgp100 for three hours and then treated with 10μM PI3K inhibitors. Phosphorylation was analyzed at 2, 5 and 10 minutes post-inhibitor addition. A) Representative flow plots of $CD8^+$ T cell phosphorylation of $STAT5^{Y694}$ and S6 $protein^{S235/236}$ 2 minutes post inhibitor addition. B) Kinetics of phosphorylation before and after inhibitor addition comparing vehicle and PI3K inhibitor-treated cells. Data analyzed by unpaired two-tail T tests at each time point, n=6 mice/group from two independent studies. All data represent the mean ± the standard deviation with statistical significance as $p<0.05$ *, $p<0.01$ **, $p<0.001$ *** and $p<0.0001$ ****.

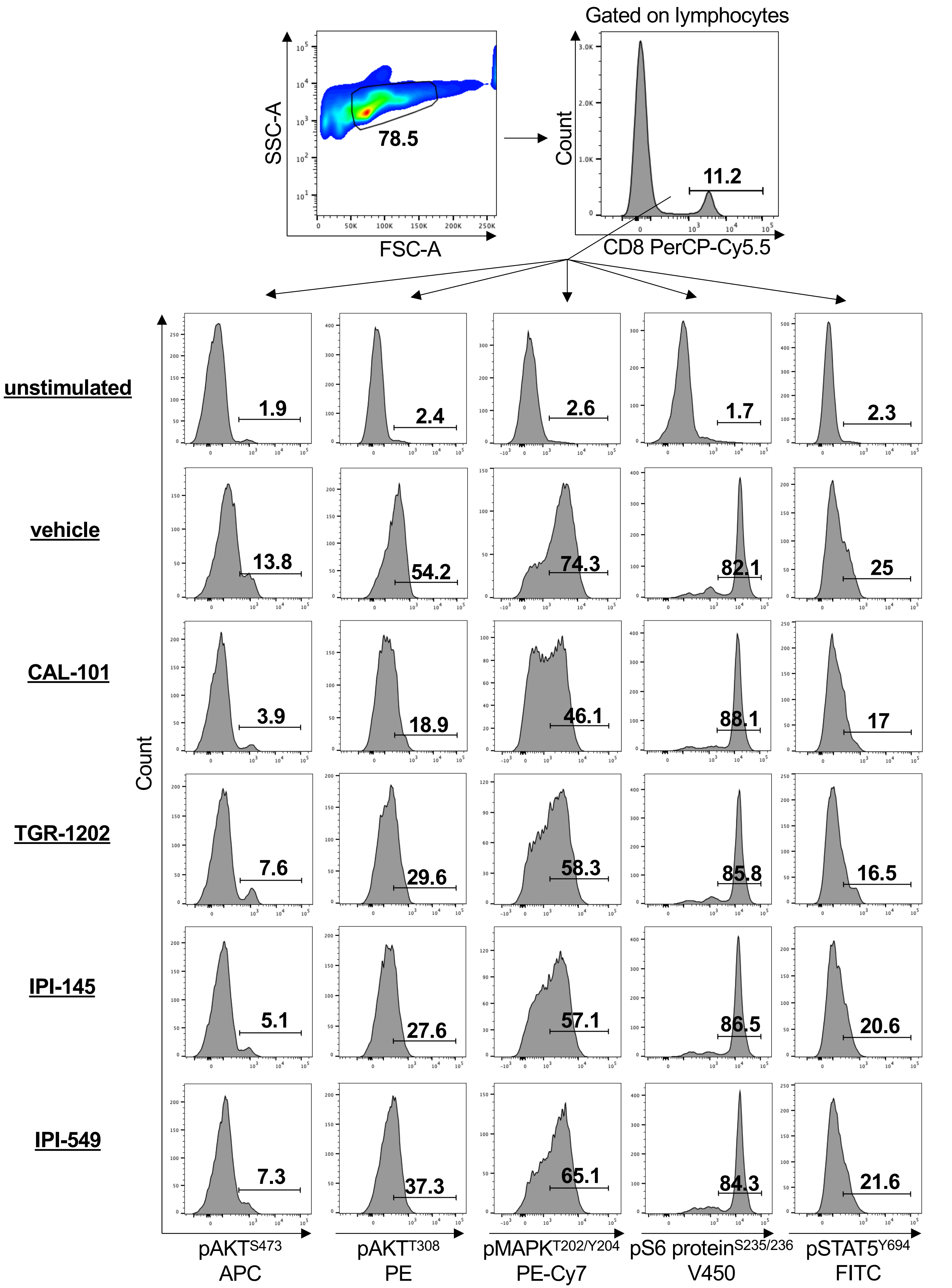

Figure S2: Representative gating scheme for the phosphorylation of signaling mediators downstream of TCR signaling in $CD8^+$ T cells.

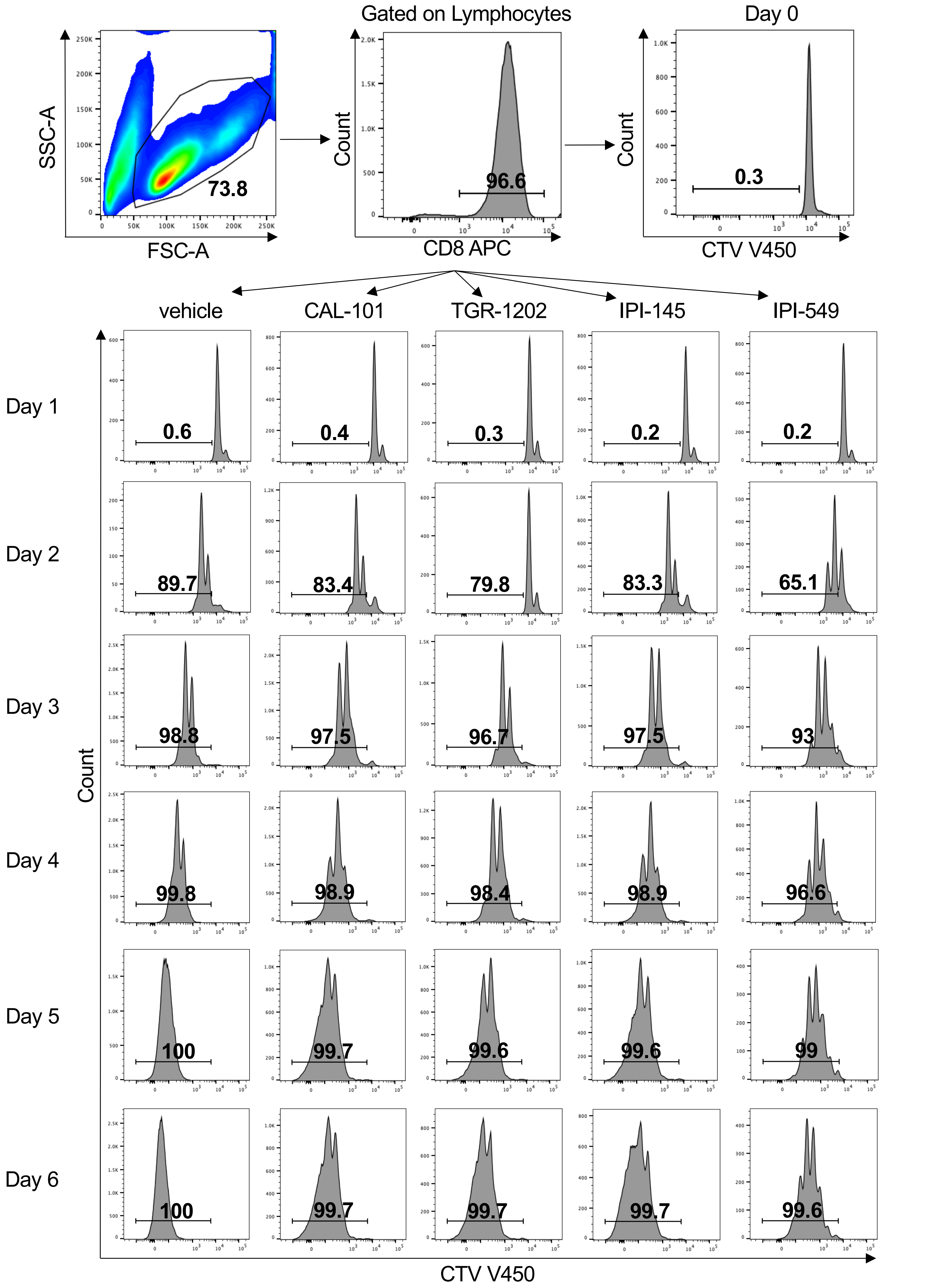

Figure S3: Representative gating scheme for cell trace violet staining of CD8⁺ T cells from Day 0 to Day 6.

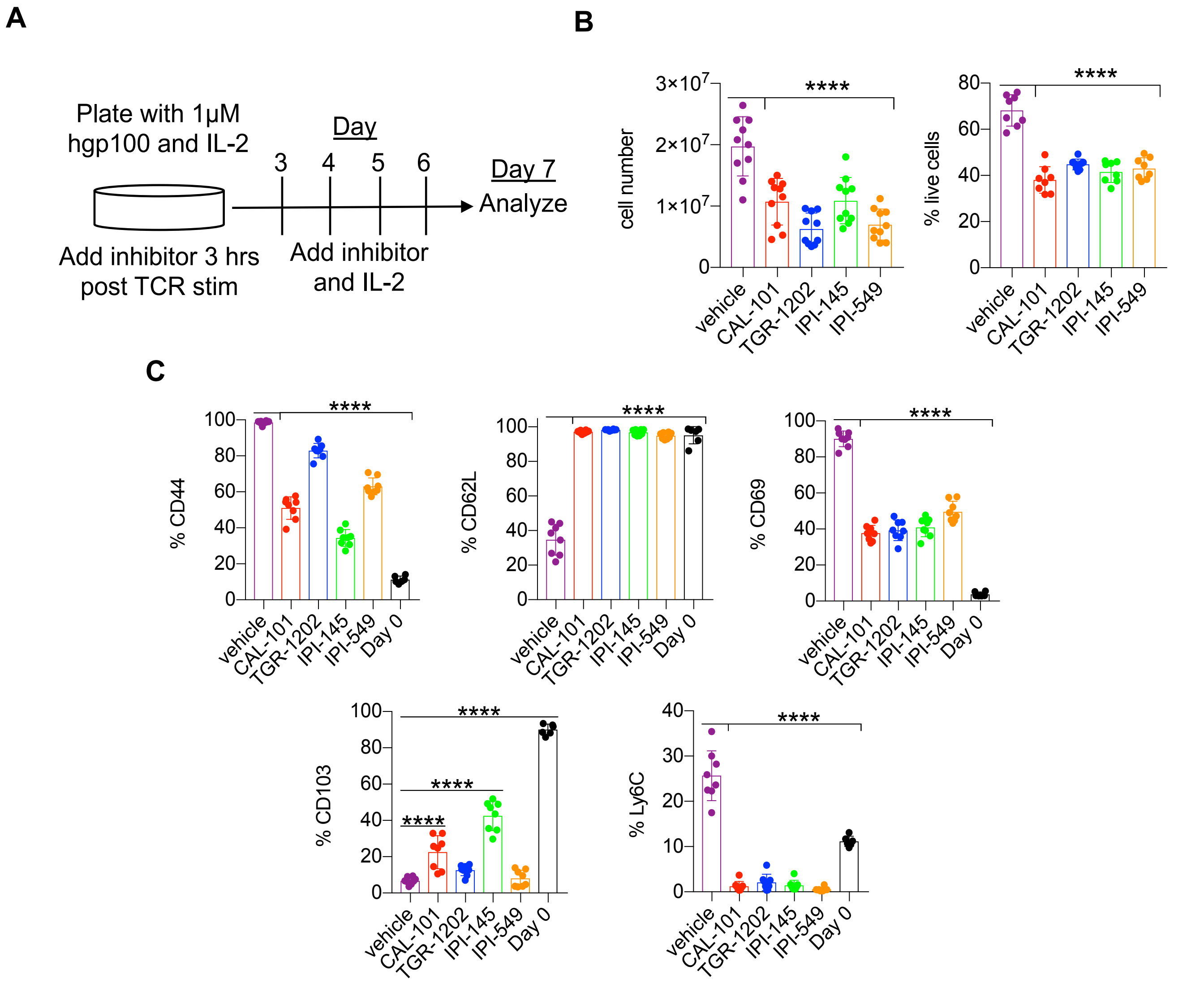

Figure S4: Reduced expansion and activation of CD8⁺ T cells with *ex vivo* PI3Kγ and/or PI3Kδ inhibition. Pmel-1 splenocytes were stimulated with hgp100 and expanded in the presence or absence of PI3K inhibitors and IL-2 for one week. A) Culture schema of *ex vivo* expansion of pmel-1 CD8⁺ T cells. Cell culture yield and viability (B) and expression of activation markers on CD8⁺ T cells from unstimulated Day 0 naïve and Day 7 expanded cells (C). Data analyzed by one-way ANOVA with Tukey's multiple comparisons, Day 0 n=6 mice and Day 7 n=8-10 mice/group from two independent experiments. All bars represent the mean ± the standard deviation with statistical significance as $p<0.05$ *, $p<0.01$ **, $p<0.001$ *** and $p<0.0001$ ****.

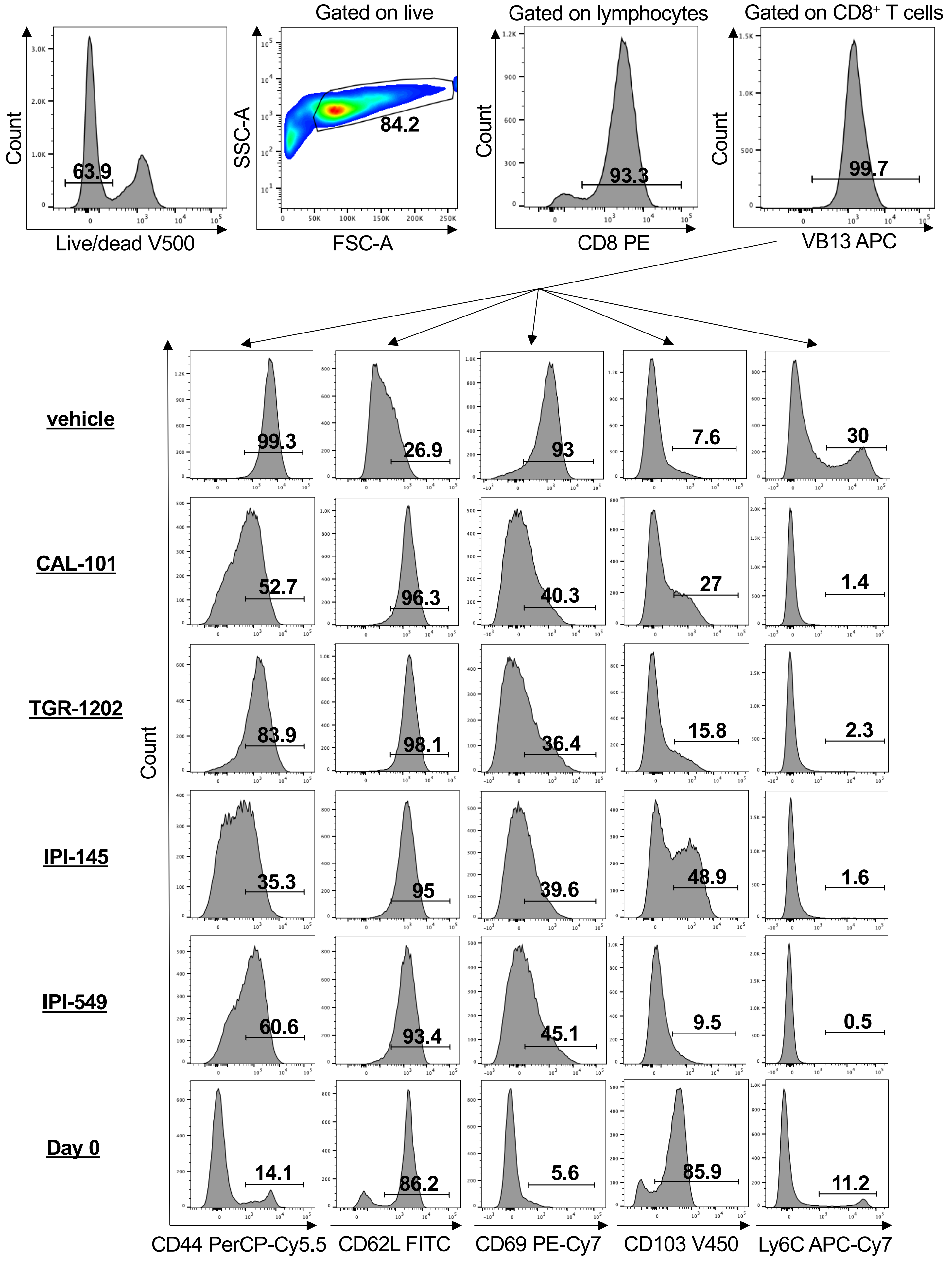

Figure S5: Representative gating scheme of CD8⁺ T cell surface marker expression on Day 7 of expansion.

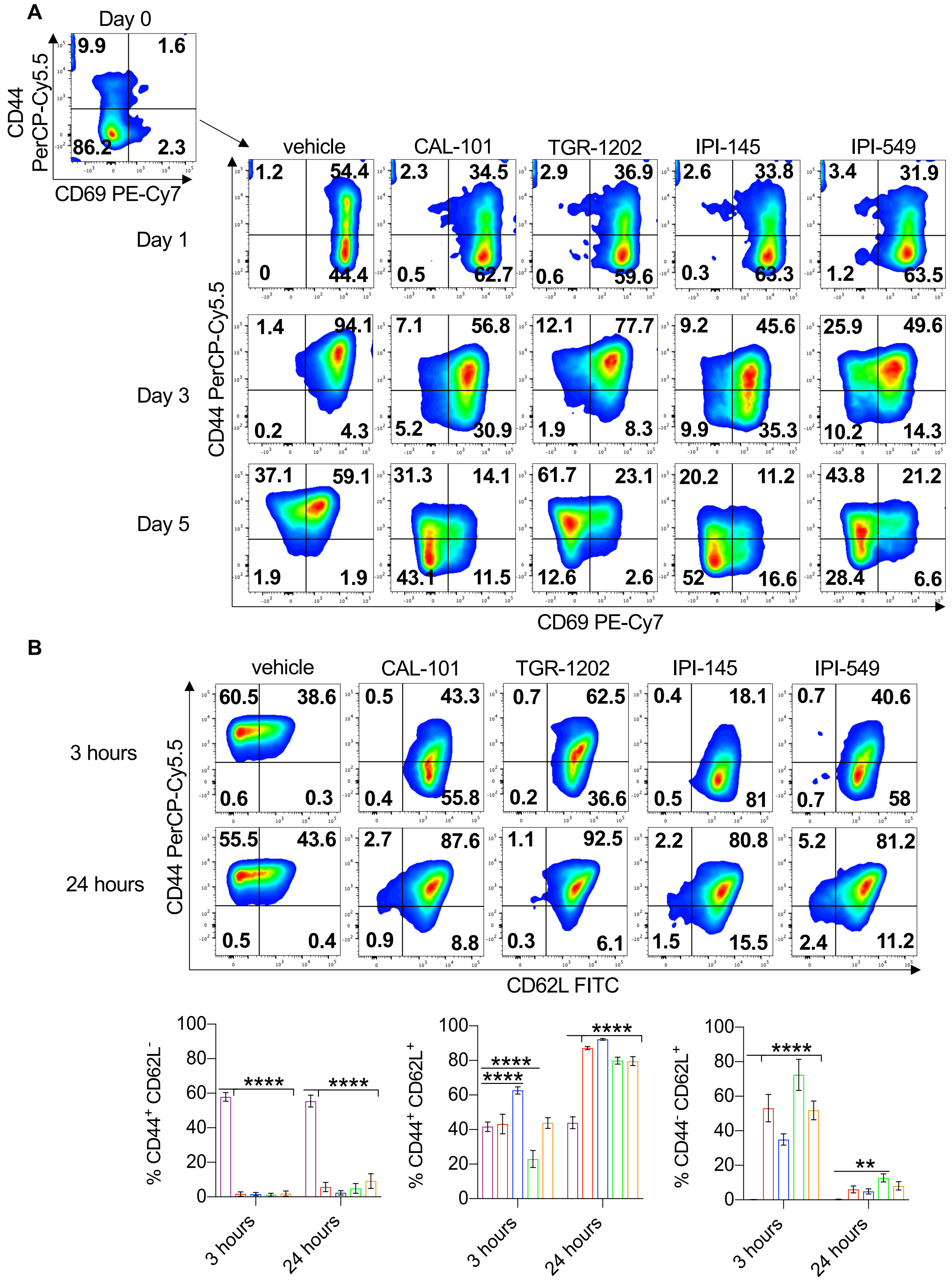

A
Day 0
CD44 PerCP-Cy5.5
CD69 PE-Cy7
9.9
1.6
86.2
2.3
vehicle
CAL-101
TGR-1202
IPI-145
IPI-549
Day 1
Day 3
Day 5
CD44 PerCP-Cy5.5
CD69 PE-Cy7
1.2
54.4
0
44.4
2.3
34.5
0.5
62.7
2.9
36.9
0.6
59.6
2.6
33.8
0.3
63.3
3.4
31.9
1.2
63.5
1.4
94.1
0.2
4.3
7.1
56.8
5.2
30.9
12.1
77.7
1.9
8.3
9.2
45.6
9.9
35.3
25.9
49.6
10.2
14.3
37.1
59.1
1.9
1.9
31.3
14.1
43.1
11.5
61.7
23.1
12.6
2.6
20.2
11.2
52
16.6
43.8
21.2
28.4
6.6
B
vehicle
CAL-101
TGR-1202
IPI-145
IPI-549
3 hours
24 hours
CD44 PerCP-Cy5.5
CD62L FITC
60.5
38.6
0.6
0.3
0.5
43.3
0.4
55.8
0.7
62.5
0.2
36.6
0.4
18.1
0.5
81
0.7
40.6
0.7
58
55.5
43.6
0.5
0.4
2.7
87.6
0.9
8.8
1.1
92.5
0.3
6.1
2.2
80.8
1.5
15.5
5.2
81.2
2.4
11.2
% CD44+ CD62L-
% CD44+ CD62L+
% CD44- CD62L+
****
**
3 hours
24 hours

Figure S6: PI3Kγ and/or PI3Kδ inhibition during initial T cell priming reduces T cell differentiation. A) Representative flow plots of pmel-1 splenocyte activation marked CD44 and CD69 from Days 1, 3, and 5 in the presence or absence of respective PI3K inhibitors, from three independent experiments. B) Pmel-1 splenocytes were stimulated with hgp100 and expanded for one week in the presence or absence of PI3K inhibitors added 3 or 24 hours post TCR stimulation with IL-2. Representative flow plots (top) and expression of T cell memory subsets (bottom) on Day 7 of expansion. Data analyzed by one-way ANOVA with Tukey's multiple comparisons, n=4 mice/group from two independent experiments. All bars represent the mean ± the standard deviation with statistical significance as $p<0.05$ *, $p<0.01$ **, $p<0.001$ *** and $p<0.0001$ ****.

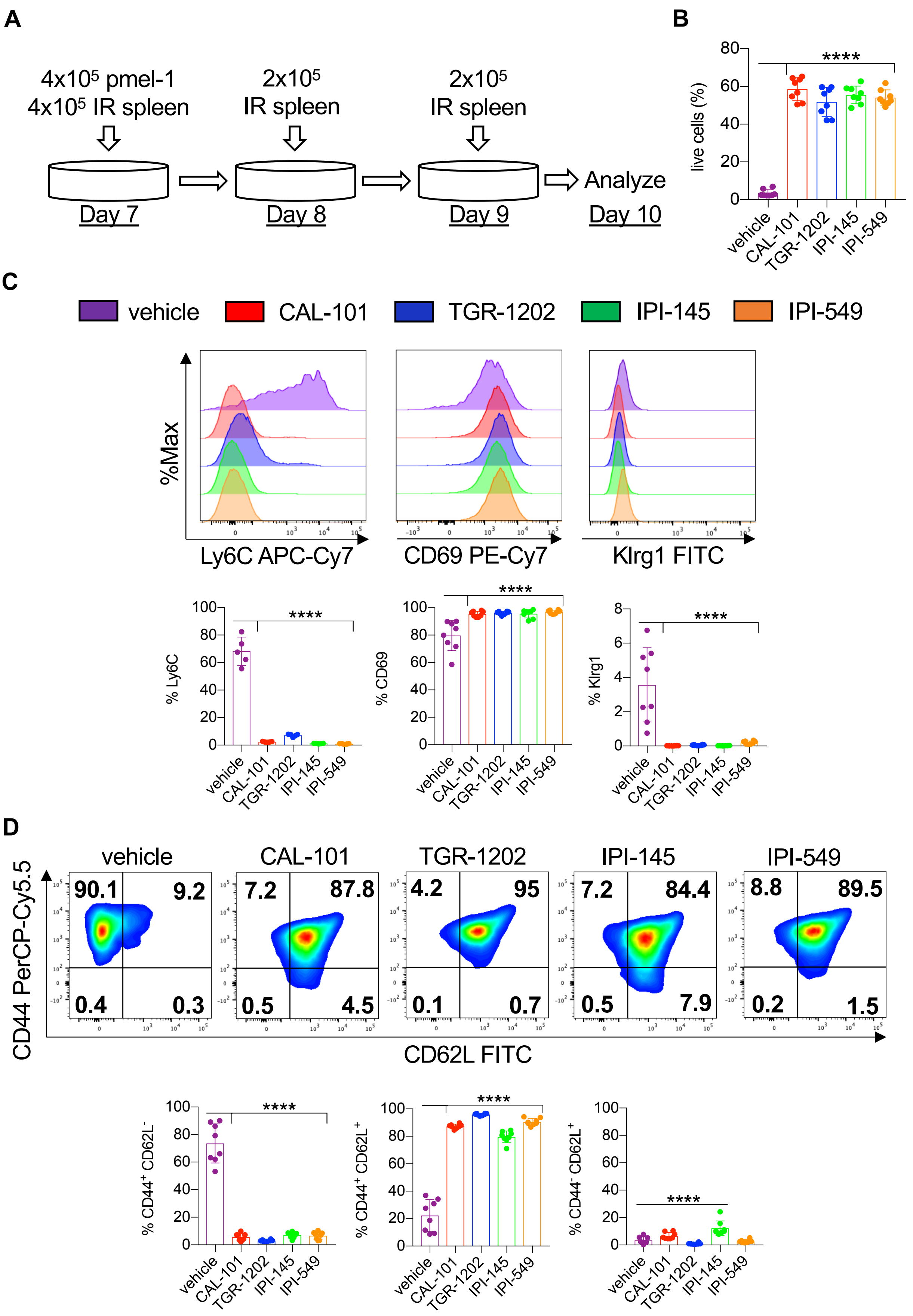

A
4x10⁵ pmel-1
4x10⁵ IR spleen
2x10⁵
IR spleen
2x10⁵
IR spleen
Day 7
Day 8
Day 9
Analyze
Day 10
B
live cells (%)
****
vehicle
CAL-101
TGR-1202
IPI-145
IPI-549
C
vehicle
CAL-101
TGR-1202
IPI-145
IPI-549
%Max
Ly6C APC-Cy7
CD69 PE-Cy7
Klrg1 FITC
% Ly6C
% CD69
% Klrg1
****
D
vehicle
CAL-101
TGR-1202
IPI-145
IPI-549
CD44 PerCP-Cy5.5
CD62L FITC
90.1
9.2
0.4
0.3
7.2
87.8
0.5
4.5
4.2
95
0.1
0.7
7.2
84.4
0.5
7.9
8.8
89.5
0.2
1.5
% CD44+ CD62L-
% CD44+ CD62L+
% CD44- CD62L+
****

Figure S7: $CD8^+$ T cells treated with PI3K inhibitors resist exhaustion during chronic antigen stimulation. $CD8^+$ pmel-1 cells were expanded for one week in the presence or absence of PI3K inhibitors and received chronic stimulation with hgp100 on Day 7, 8 and 9 of culture in the presence of PI3K inhibitors. A) Experimental schema of *ex vivo* chronic stimulation of $CD8^+$ T cells. B) Culture viability of vehicle and PI3K inhibitor-treated cells. Representative flow plots (top) and expression (bottom) of activation markers (C) and memory differentiation (D) on Day 10 of culture. Data analyzed by one-way ANOVA with Tukey's multiple comparisons, n=8 mice/group from two independent experiments. All bars represent the mean ± the standard deviation with statistical significance as $p<0.05$ *, $p<0.01$ **, $p<0.001$ *** and $p<0.0001$ ****.

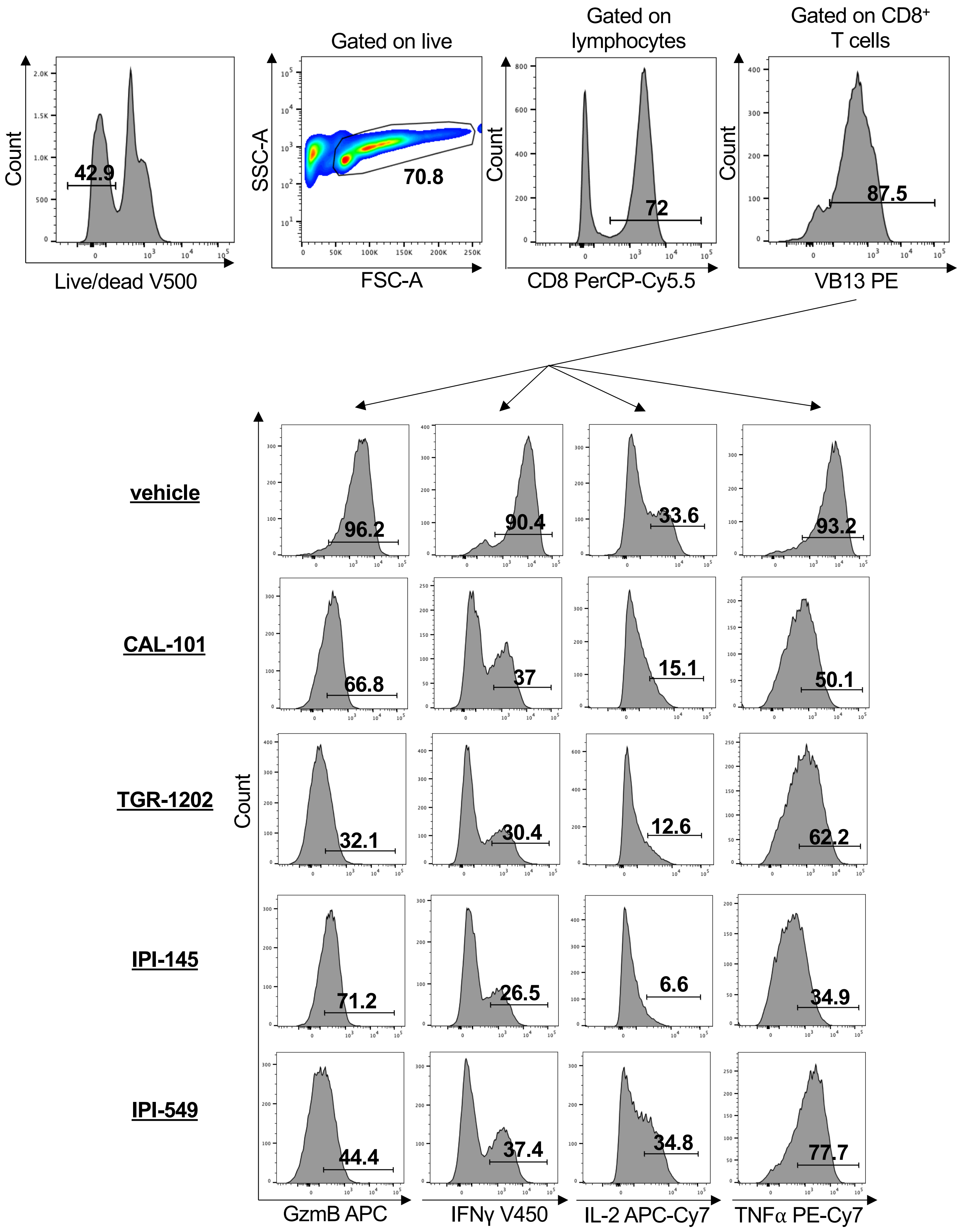

Figure S8: Representative gating scheme of intracellular cytokine staining of Day 8 CD8^{+} T cells.

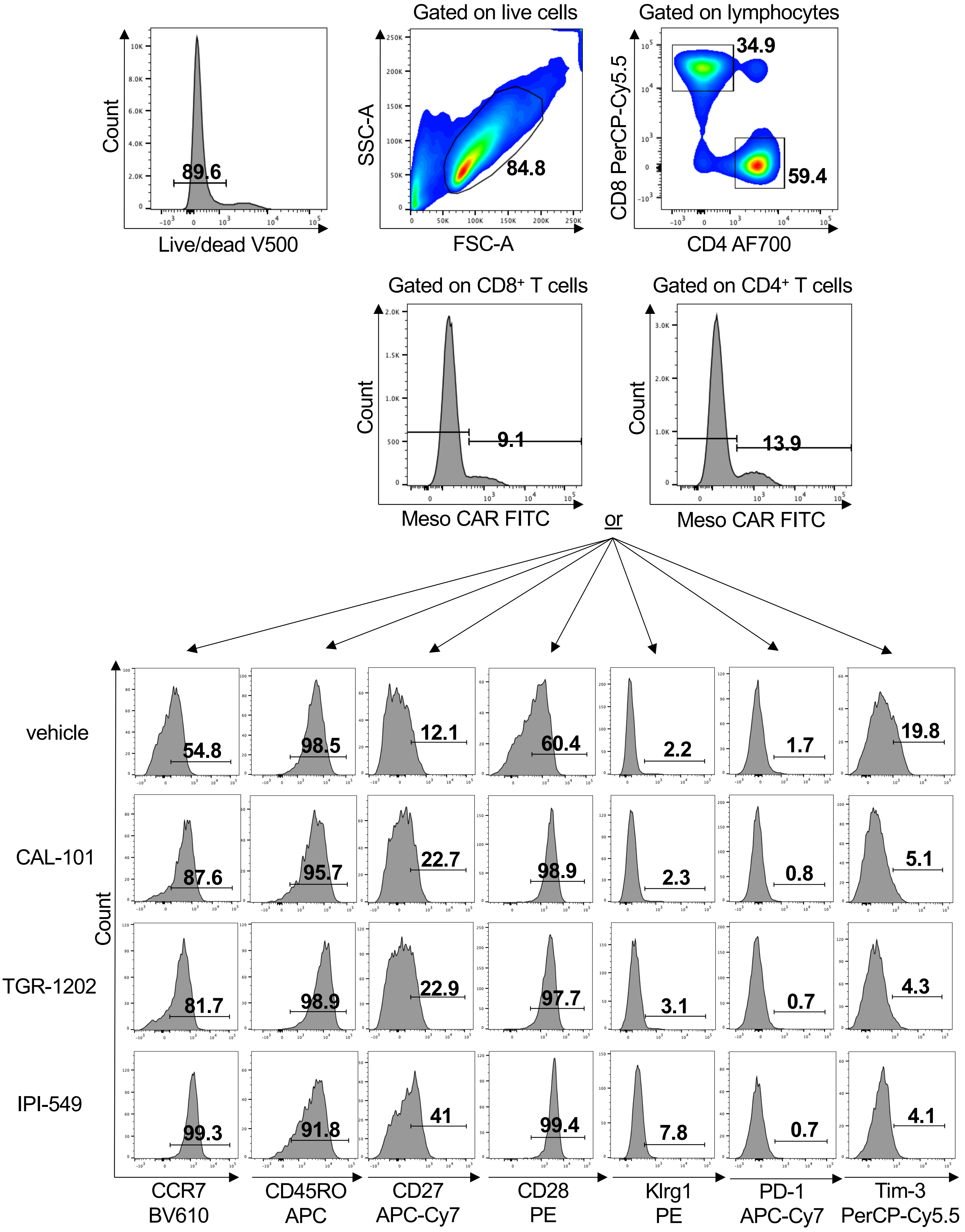

Figure S9: Representative gating scheme of surface markers on human CAR T cells on Day 7 of expansion.

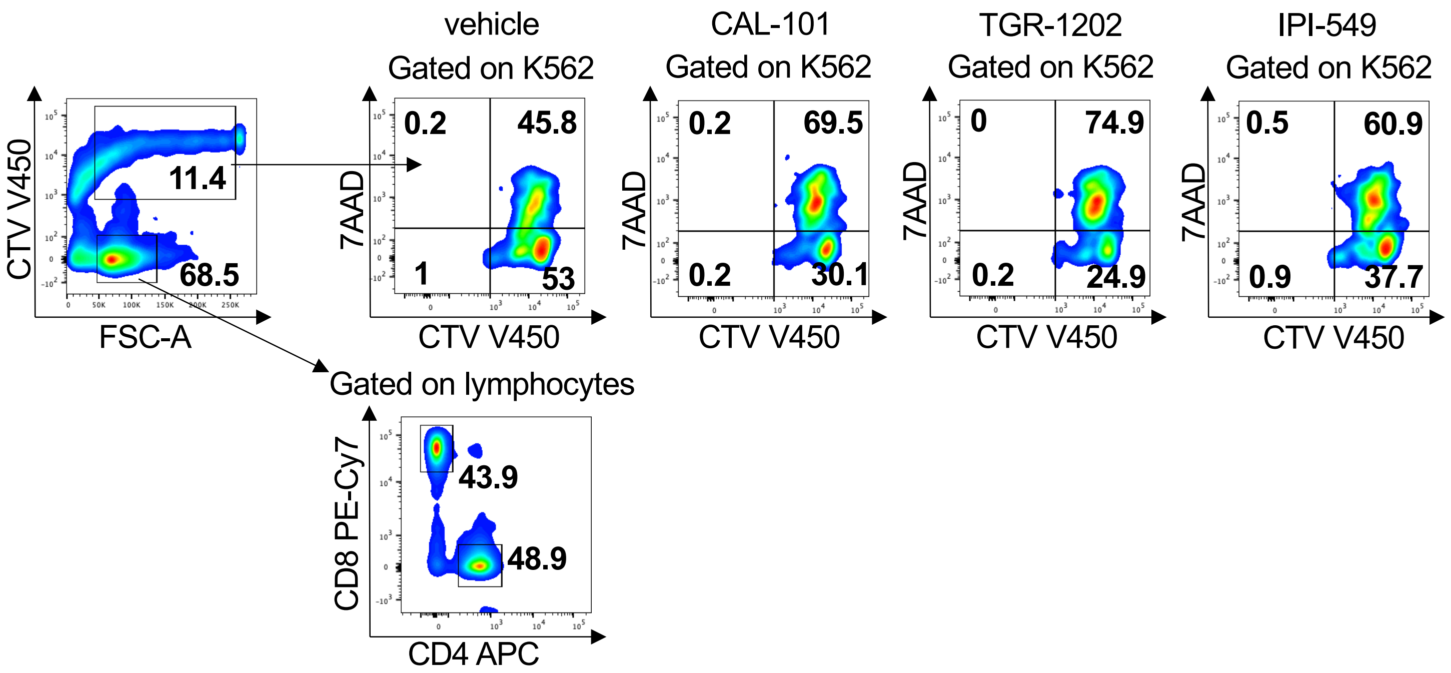

Figure S10: Representative gating scheme of the *in vitro* CAR cytotoxicity assay on Day 15.
